# Outer Membrane Disruption Overcomes Intrinsic, Acquired, and Spontaneous Antibiotic Resistance

**DOI:** 10.1128/mBio.01615-20

**Published:** 2020-09-22

**Authors:** Craig R. MacNair, Eric D. Brown

**Affiliations:** a Department of Biochemistry and Biomedical Sciences, McMaster University, Hamilton, Ontario, Canada; b Michael G. DeGroote Institute of Infectious Disease Research, McMaster University, Hamilton, Ontario, Canada; University of Rochester

**Keywords:** Gram-negative, antibiotic adjuvant, antibiotic resistance, outer membrane

## Abstract

Disruption of the outer membrane (OM) barrier allows for the entry of otherwise inactive antimicrobials into Gram-negative pathogens. Numerous efforts to implement this approach have identified a large number of OM perturbants that sensitize Gram-negative bacteria to many clinically available Gram-positive active antibiotics. However, there is a dearth of investigation into the strengths and limitations of this therapeutic strategy, with an overwhelming focus on characterization of individual potentiator molecules. Herein, we look to explore the utility of exploiting OM perturbation to sensitize Gram-negative pathogens to otherwise inactive antimicrobials. We identify the ability of OM disruption to change the rules of Gram-negative entry, overcome preexisting and spontaneous resistance, and impact biofilm formation. Disruption of the OM expands the threshold of hydrophobicity compatible with Gram-negative activity to include hydrophobic molecules. We demonstrate that while resistance to Gram-positive active antibiotics is surprisingly common in Gram-negative pathogens, OM perturbation overcomes many antibiotic inactivation determinants. Further, we find that OM perturbation reduces the rate of spontaneous resistance to rifampicin and impairs biofilm formation. Together, these data suggest that OM disruption overcomes many of the traditional hurdles encountered during antibiotic treatment and is a high priority approach for further development.

## Abstract (precis)

The spread of antibiotic resistance is an urgent threat to global health that necessitates new therapeutics. Treatments for Gram-negative pathogens are particularly challenging to identify due to the robust outer membrane permeability barrier in these organisms. Recent discovery efforts have attempted to overcome this hurdle by disrupting the outer membrane using chemical perturbants and have yielded several new peptides and small molecules that allow the entry of otherwise inactive antimicrobials. However, a comprehensive investigation into the strengths and limitations of outer membrane perturbants as antibiotic partners is currently lacking. Herein, we interrogate the interaction between outer membrane perturbation and several common impediments to effective antibiotic use. Interestingly, we discover that outer membrane disruption is able to overcome intrinsic, spontaneous, and acquired antibiotic resistance in Gram-negative bacteria, meriting increased attention toward this approach.

## INTRODUCTION

Increasing the arsenal of available antibiotics is paramount to addressing the growing resistance crisis (1, 2). Encouraging progress has been made in the treatment of Gram-positive pathogens (3), with two new antibiotic classes, cyclic lipopeptides (daptomycin) and oxazolidinones (linezolid), introduced within the last 20 years. Additionally, two novel Gram-positive active antibiotics are currently in clinical trials (3). Unfortunately, antibiotic development for Gram-negative bacteria has remained stagnant. The last novel class of Gram-negative active antibiotics, the quinolones, were introduced into the clinic over 50 years ago (4), and none are currently in the clinical pipeline (3).

The failure to develop antibiotics with Gram-negative activity can largely be attributed to the inability of small molecules to accumulate within these bacteria (5). All Gram-negative bacteria are protected from toxic stressors by an outer membrane (OM) that reduces compound influx into the cell (6). The OM is an asymmetric bilayer composed of lipopolysaccharides (LPS) in the outer leaflet and phospholipids in the inner leaflet (7) and is found uniquely within Gram-negative bacteria. Tight packing of LPS and an overall negative charge act to exclude most large and hydrophobic molecules (8). Permeability has mostly limited Gram-negative active antimicrobials to those capable of traversing through porins (9).

The target of many Gram-positive active antibiotics is present in Gram-negative bacteria; arguably, the only barrier to their activity against Gram-negative bacteria is their inability to permeate the OM. Indeed, strains of Escherichia coli with compromised OM or efflux capability are highly sensitized to many of these traditionally Gram-positive active antibiotics (10–12). Over the last 30 years, antibiotic discovery efforts have attempted to increase the intracellular concentration of Gram-positive active antibiotics in Gram-negative pathogens through a variety of approaches, including inhibition of efflux machinery, medicinal chemistry efforts, and chemical perturbation of the OM.

Antibiotic activity against Gram-negative bacteria is primarily restricted to compounds with high polarity and a molecular weight (MW) of less than 600 Da (5). Recent work has expanded these “rules” of Gram-negative entry identifying molecules that are rigid and flat and contain positive charge to be more compatible with passage through porins (13, 14). These concepts have been applied in medicinal chemistry efforts to alter Gram-positive active antibiotics for Gram-negative activity by developing analogues that better adhere to the parameters of Gram-negative entry. This approach, while promising, is limited to scaffolds amenable to modification without losing affinity for their intracellular target and may have a detrimental impact on otherwise favorable pharmacological properties of these drugs. An alternative approach is the direct perturbation of the OM barrier, which facilitates the entry of many Gram-positive active antibiotics into Gram-negative pathogens (15). Indeed, an approved OM perturbant used alongside clinically proven Gram-positive active antibiotics would immediately expand the arsenal of available treatments for Gram-negative pathogens (16).

The unique properties of LPS make the OM distinct from eukaryotic membranes and an exploitable bacterial target. OM-perturbing peptides (17, 18), small molecules (10), and chelators (7, 19) disrupt the divalent cation bridges that stabilize LPS. Disruption of the OM sensitizes bacteria to many Gram-positive antibiotics (15), an approach that has been rigorously studied with the OM perturbant, polymyxin B nonapeptide (PMBN) since its discovery in 1983 (20). The rise of antibiotic resistance in Gram-negative pathogens has brought renewed attention to this area. Indeed, a wealth of recent work shows that OM perturbants in combination with traditionally Gram-positive active antibiotics can successfully treat murine infection models of Acinetobacter baumannii (10, 21), Klebsiella pneumoniae (22), and Escherichia coli (23). In 2017, the first OM perturbant SPR741, a derivative of PMBN (24), completed phase Ia and Ib clinical trials with a promising pharmacokinetic and tolerability profile (25). It is currently unknown if this potent OM perturbant will advance into phase II trials.

Despite the growing number of promising OM perturbants, questions remain on the potential of this antibiotic strategy (26). As a combination approach, growth inhibition relies on the activity of both the OM disruptor and partner antibiotic, which may increase susceptibility to resistance development (27). Additionally, a high abundance of resistant elements for Gram-positive active antibiotics are present in Gram-negative bacteria (28). Therefore, while OM perturbation may allow entry of many Gram-positive active antibiotics into Gram-negative pathogens, growth inhibition might be ineffective due to preexisting resistance. In addition to horizontally acquired resistance, OM perturbation is likely to encounter many of the same challenges that plague Gram-negative antibiotic treatment, including spontaneous resistance development and biofilms. Herein, we look to interrogate the potential of OM perturbation as an approach in antibiotic combination treatment. We first investigate how OM disruption changes the rules of Gram-negative entry, identifying a significant expansion to the threshold of hydrophobicity compatible with Gram-negative activity. We next uncover the ability for OM perturbation to render many antibiotic inactivation resistance elements ineffective, as well as decrease the development of spontaneous resistance. Finally, we explore the ability of OM disruption to attenuate biofilm formation. Overall, we find that OM perturbation overcomes many of the perceived hurdles to its clinical implementation, warranting increased attention toward this highly rewarding approach.

## RESULTS

### OM perturbation increases the range of hydrophobicity compatible with Gram-negative entry.

Several diverse stressors are known to permeabilize the OM, including magnesium limitation (29), chelators (19), peptides (18), small organic compounds (10), and genetic perturbations (30). We first sought to investigate whether antibiotic sensitivity in E. coli varies with the type of OM perturbant used, focusing our efforts on five potentiators covering the major categories of known OM disruptors: the chelator (ethylenediaminetetraacetic acid [EDTA]), small molecule (pentamidine), peptides (colistin and SPR741), and deletion of the *waaC* gene. While structurally distinct, the perturbants EDTA, pentamidine, colistin, and SPR741 all increase OM permeability by disrupting the cation bridging between LPS molecules. Deleting *waaC* in E. coli compromises the OM by ablating the heptosyltransferase that adds the first heptose sugar onto the Kdo_2_ moiety of LPS inner core, truncating LPS structure (31).

**FIG 1:**
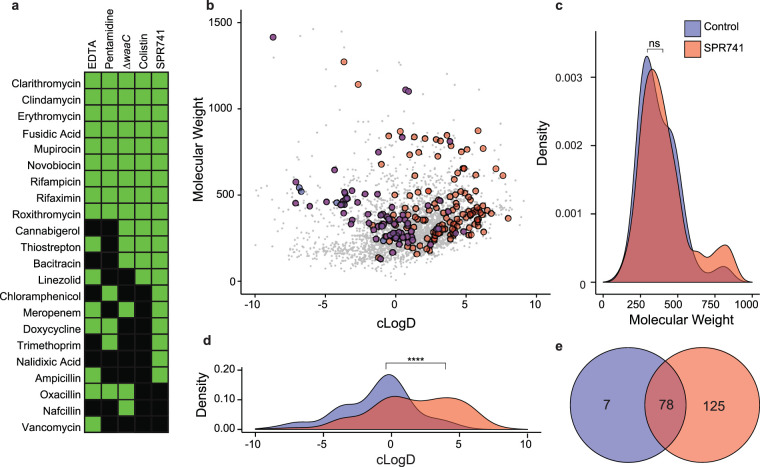
Identifying changes in permeability by outer membrane perturbation. (a) Heat map showing antimicrobials potentiated (reduction in MIC >4-fold shown in green) or unaffected (reduction in MIC ≤ 4-fold shown in black) by five OM-perturbing conditions. (b) Physicochemical space of 3,645 compounds screened for bacterial growth inhibition, visualized by molecular weight and calculated logD (cLogD) at pH 7.4. Compounds are colored by growth-inhibitory activity, E. coli control (blue), E. coli with SPR741 (red), no activity in either condition (gray) and activity in both conditions (purple). (c and d) Density plots of molecular weight and cLogD for growth-inhibitory compounds in the E. coli control (blue) and SPR741 condition (red). SPR741 significantly alters the hydrophobicity of compounds compatible with growth inhibition (****, *P* < 0.0001 by the Kolmogorov-Smirnov test) (d) but not molecular weight (c) (*P* > 0.05 by the Kolmogorov-Smirnov test). ns, not significant. (e) Venn diagram showing the number and overlap of compounds with growth-inhibitory activity in the E. coli control (blue) and SPR741 condition (red).

We screened a panel of 43 antibiotics to measure their degree of potentiation alongside these five OM perturbants. Compounds were considered potentiated if the MIC was reduced >4-fold compared to a no-treatment control (Fig. 1a; see also Table S1 in the supplemental material). SPR741 potentiated the highest number of antibiotics, followed by EDTA, Δ*waaC*, colistin, and pentamidine. Of the 43 antibiotics tested, 22 were potentiated by at least one type of OM perturbant. As previously reported, hydrophobic antibiotics were highly compatible with potentiation (15). Nine large, hydrophobic, traditionally Gram-positive active antibiotics (novobiocin, fusidic acid, mupirocin, clarithromycin, erythromycin, roxithromycin, clindamycin, rifampicin, and rifaximin) were potentiated by all five potentiators tested. We found that potentiation was often conserved between OM perturbants with potentiation in three or more conditions observed for 16 of 22 drugs, with some exceptions. For example, the MIC of vancomycin is reduced 32-fold by EDTA but ≤4-fold for all other probes. Additionally, we noted a complete absence of potentiation for 21 of 43 drugs, a subset that mostly comprised Gram-negative active antibiotics, such as the fluoroquinolones, tetracyclines, aminoglycosides, and β-lactams (Table S1). Notably, potentiation of β-lactams appears to be compound specific as we observed moderate potentiation (<10-fold) in at least one OM perturbant with four of seven β-lactams tested. Taken together, these data indicate moderate variability in antibiotic potentiation with respect to the OM perturbant. However, we observe striking conservation in the potentiation of macrolides, rifamycins, and other hydrophobic Gram-positive active antibiotics, irrespective of the source of OM disruption.

10.1128/mBio.01615-20.6 TABLE S1 Fold reduction of MIC in the presence of OM perturbants compared to an untreated control. Data are representative of at least two biological replicates. Download Table S1, XLSX file, 0.01 MB. Copyright © 2020 MacNair and Brown. 2020 MacNair and Brown This content is distributed under the terms of the Creative Commons Attribution 4.0 International license.

Next, we looked to investigate how OM perturbation may expand the thresholds of MW and hydrophobicity compatible with Gram-negative activity, as entry through OM porins is typically restricted to small, hydrophilic compounds with a MW of less than 600 Da (9). To this end, we screened a library of 3,645 known bioactive compounds that included off-patent drugs, natural products, and other biologically active compounds in four conditions: E. coli, E. coli with SPR741, E. coli Δ*waaC*, and methicillin-resistant Staphylococcus aureus (MRSA). SPR741 was selected from the four chemical probes because it potentiated the highest number of antibiotics (Fig. 1a) and is currently the closest OM perturbant to clinical implementation. We anticipate that potentiation by SPR741 would highly correlate with other OM perturbants.

We calculated MW and lipophilicity (calculated partition coefficient [cLogD] at pH 7.4) for all 3,645 screening compounds (Fig. 1b to d), and classified those that reduced normalized growth below 50% as growth inhibitory (see Fig. S1 in the supplemental material). From this, we found that OM perturbation increased the number of growth-inhibitory compounds from 85 in E. coli alone to 203 in E. coli with SPR741, 78 of which overlap between the two conditions (Fig. 1e). Compounds with growth-inhibitory activity against E. coli alone largely adhered to the previously established rules of MW compatible with Gram-negative permeability with a mean MW of 406.6, and 92% of compounds less than 600 Da (Fig. 1c). Comparatively, an analysis of the 203 inhibitors with growth-inhibitory activity against E. coli with SPR741 revealed a trend toward a larger MW (mean of 437.9, but not statistically different from 406.6), with 87% of inhibitors less than 600 Da (Fig. 1c). We note that the enrichment of our library for compounds <600 Da (Fig. 1c) may hinder our analysis of MW. Nevertheless, the addition of SPR741 significantly expanded the range of cLogD compatible with antimicrobial activity toward more hydrophobic compounds (Fig. 1d). The average cLogD of compounds inhibiting E. coli growth was −1.34 compared to 1.57 in the presence of SPR741, an approximately 800-fold increase. Indeed, of the 125 compounds with growth-inhibitory activity dependent upon the presence of SPR741, 87% are considered hydrophobic (cLogD > 0).

10.1128/mBio.01615-20.1 FIG S1 Replicate plots of normalized growth for the primary screen of 3,645 compounds in four conditions: E. coli, E. coli with SPR741, E. coli Δ*waaC*, and methicillin-resistant Staphylococcus aureus (MRSA). Compounds inhibiting growth below 0.5 normalized growth in either replicate are considered active (red). Download FIG S1, JPG file, 1.9 MB. Copyright © 2020 MacNair and Brown. 2020 MacNair and Brown This content is distributed under the terms of the Creative Commons Attribution 4.0 International license.

The use of a Δ*waaC* strain of E. coli phenocopied the expansion of growth-inhibitory compounds in the presence of SPR741. Growth inhibition against Δ*waaC* was observed in 108 compounds, which show a nonsignificant increase in average MW but a significant increase in lipophilicity, compared to those compounds active against E. coli (Fig. S2).

10.1128/mBio.01615-20.2 FIG S2 Physicochemical space of compounds screened for bacterial growth inhibition, visualized by molecular weight and calculated logD (cLogD) at pH 7.4 (a, c, and d). Compounds are colored by growth-inhibitory activity as follows: no activity (gray), activity in only the E. coli control (blue), activity in E. coli Δ*waaC* only (yellow), and activity in both conditions (green). (a and d) Density plots of molecular weight and cLogD for growth-inhibitory compounds. Δ*waaC* significantly alters the hydrophobicity of compounds compatible with growth inhibition (*P* < 0.0001, Kolmogorov-Smirnov test) but not molecular weight (*P* > 0.05, Kolmogorov-Smirnov test). (b) Venn diagram showing the number and overlap of compounds with growth-inhibitory activity in the E. coli control (blue) and Δ*waaC* condition (yellow). Download FIG S2, EPS file, 2.5 MB. Copyright © 2020 MacNair and Brown. 2020 MacNair and Brown This content is distributed under the terms of the Creative Commons Attribution 4.0 International license.

Finally, we looked to compare whether OM perturbation recapitulated the range of physicochemical properties compatible with activity against the Gram-positive pathogen MRSA. Growth inhibition was observed in 177 compounds, of which 122 overlap with those active in the presence of E. coli with SPR741 (Fig. S3a to d). Compounds inhibitory to S. aureus had an average MW of 479.6 and cLogD of 1.32. There was no significant difference in the MW or hydrophobicity of active compounds when comparing MRSA and E. coli in the presence of SPR741 or the deletion of Δ*waaC* (Fig. S3 and S4). Together, these results indicate the ability for OM disruption to increase the range of hydrophobicity compatible with growth inhibition, similar to that observed for Gram-positive bacteria.

10.1128/mBio.01615-20.3 FIG S3 Physicochemical space of compounds screened for bacterial growth inhibition, visualized by molecular weight and calculated logD (cLogD) at pH 7.4 (a, c, and d). Compounds are colored by growth-inhibitory activity as follows: no activity (gray), activity in S. aureus (yellow), activity for E. coli with SPR741 only (red), and activity in both conditions (orange). (a and d) Density plots of molecular weight and cLogD for growth-inhibitory compounds. No significant difference in hydrophobicity or molecular weight (*P* > 0.05, Kolmogorov-Smirnov test) was observed. (b) Venn diagram showing the number and overlap of compounds with growth-inhibitory activity in E. coli with SPR741 (red) and S. aureus (yellow). Download FIG S3, EPS file, 2.4 MB. Copyright © 2020 MacNair and Brown. 2020 MacNair and Brown This content is distributed under the terms of the Creative Commons Attribution 4.0 International license.

10.1128/mBio.01615-20.4 FIG S4 Physicochemical space of compounds screened for bacterial growth inhibition, visualized by molecular weight and calculated logD (cLogD) at pH 7.4 (a, c, and d). Compounds are colored by growth-inhibitory activity as follows: no activity (gray), activity in only E. coli Δ*waaC* (yellow), activity in S. aureus (pink), and activity in both conditions (orange). (a and d) Density plots of molecular weight and cLogD for growth-inhibitory compounds. No significant difference in cLogD or molecular weight was observed between these conditions (*P* > 0.05, Kolmogorov-Smirnov test). (b) Venn diagram showing the number and overlap of compounds with growth-inhibitory activity in the E. coli Δ*waaC* (yellow) and S. aureus (pink) condition. Download FIG S4, EPS file, 2.6 MB. Copyright © 2020 MacNair and Brown. 2020 MacNair and Brown This content is distributed under the terms of the Creative Commons Attribution 4.0 International license.

### OM disruption overcomes antibiotic inactivation.

Perturbation of the OM sensitizes Gram-negative bacteria to a wide range of Gram-positive active antibiotics. Previous work has focused on a limited number of antibiotic classes compatible with OM perturbation—primarily rifamycins (21), aminocoumarins (10), and macrolides (22). Antibiotics in these classes are highly potentiated by all OM-disrupting probes (Fig. 1a) and are efficacious alongside OM perturbants in murine models of infection (10, 21, 22). Notably, aminocoumarin antibiotics are not currently available for clinical use, making macrolide and rifamycins the most readily available partners for a clinically approved OM perturbant. Given this, we aimed to investigate how resistance to macrolide and rifamycin antibiotics impacts potentiation by OM disruption.

**FIG 2:**
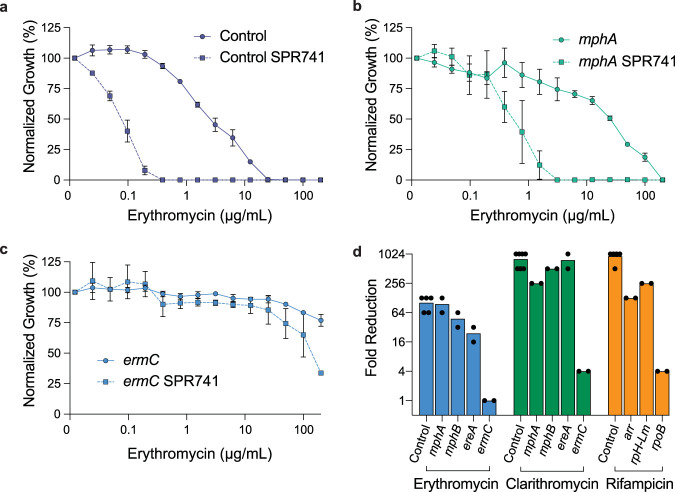
Perturbation of the outer membrane overcomes resistance by antibiotic inactivation. (a to c) Potency analysis of erythromycin in E. coli harboring plasmid control (a), *mphA* (b), or *ermC* (c) in the presence and absence of SPR741. Data shown represent the means ± standard errors of the means (SEM) (error bars) for at least two biological replicates. (d) Fold reduction of MIC by SPR741 for erythromycin, clarithromycin, and rifampicin in the presence of various resistance elements. Fold reduction is calculated by dividing the MIC of an antibiotic alone by its MIC in the presence of an OM perturbant.

We first transformed individual plasmids constitutively expressing the macrolide resistance elements *mphA* and *ermC* into E. coli and then determined the MIC of these strains to erythromycin in the presence and absence of SPR741. Perturbation of a control strain (E. coli transformed with empty vector) by SPR741 reduces the MIC for erythromycin 64-fold from 25 μg/ml to 0.39 μg/ml (Fig. 2a). Introduction of the macrolide resistance phosphatase MphA increases the MIC of erythromycin to 200 μg/ml (Fig. 2b). In this strain, OM perturbation by SPR741 reduced the MIC of erythromycin to 3.125 μg/ml (Fig. 2b), maintaining the same level of reduction (64-fold) observed in the empty vector control. Conversely, expression of *ermC*, a 23S rRNA methylation enzyme (32, 33), increases the MIC of erythromycin in E. coli to above 200 μg/ml, irrespective of the addition of SPR741 (Fig. 2c).

We next extended this analysis to several additional macrolide (*mphB* and *ereA*) and rifampicin (*arr*, *rph-Lm*, and *rpoB*) resistance elements. Using the same constitutive expression plasmid system, we determined the MIC of these strains to erythromycin, clarithromycin, or rifampicin in the presence and absence of SPR741. Here, we found that E. coli harboring the macrolide-inactivating enzymes MphB, a phosphatase, and EreA, an esterase, are susceptible to erythromycin potentiation by SPR741 with an average reduction in MIC of 48- and 24-fold, respectively (Fig. 2d). Similar results were observed for the potentiation of clarithromycin by SPR741 against E. coli expressing *mphA*, *mphE*, and *ereA* with a reduction in the MIC of clarithromycin similar to that observed in the empty vector control strain (Fig. 2d). Expression of the target-modifying resistance gene *ermC* limited potentiation of clarithromycin by SPR741, consistent with results observed for erythromycin.

Rifampicin is highly potentiated by SPR741, such that its MIC in a control E. coli strain (containing empty vector) is reduced 1,024-fold from 6.25 μg/ml to 0.006 μg/ml (Fig. 2d). We observed that E. coli strains that harbor the rifampicin inactivation enzymes Arr or Rph-Lm are significantly less susceptible to rifampicin (MIC, 400 μg/ml) but are sensitized in the presence of SPR741 (*arr*, 128-fold reduction in MIC; *rph-Lm*, 256-fold reduction in MIC) (Fig. 2d). Conversely, the introduction of a mutation in *rpoB*, which reduces the binding of rifampicin to its target, increases the MIC of rifampicin to 400 μg/ml and is mostly unaffected by SPR741 (fourfold reduction in MIC).

Last, we queried whether OM perturbation alters the efficacy of resistance elements to Gram-negative active antibiotics not highly potentiated by OM disruption. We speculated that OM perturbation might impact the function or activity of resistance enzymes beyond increasing antibiotic influx. Ten additional resistance elements were tested, covering a wide range of antibiotic classes (Fig. S5). We observed no significant reduction of MIC in strains harboring resistance with perturbation by SPR741, suggesting that these resistance elements continue to operate irrespective of OM disruption. We predict that the use of OM perturbants may overcome resistance elements but only for antibiotics where compound accumulation is limiting. Additionally, the mechanism of antibiotic resistance is vital in determining whether OM perturbation will be efficacious as we observe susceptibility in strains expressing antibiotic inactivation but not target modification.

10.1128/mBio.01615-20.5 FIG S5 Fold reduction of MIC for nonpotentiated antibiotics. Each antibiotic was tested in an E. coli vector control and E. coli harboring the specified resistance gene. Fold reduction is calculated by dividing the MIC of the antibiotic alone by its MIC in the presence of SPR741. Download FIG S5, EPS file, 1.5 MB. Copyright © 2020 MacNair and Brown. 2020 MacNair and Brown This content is distributed under the terms of the Creative Commons Attribution 4.0 International license.

### OM perturbation is efficacious against clinical E. coli isolates.

**FIG 3:**
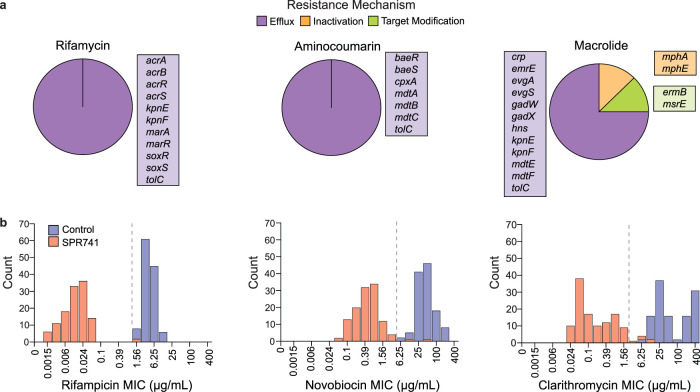
Gram-positive antibiotics are potentiated to therapeutic levels in clinical E. coli isolates by OM perturbation. (a) Resistance genes for rifamycin, aminocoumarin, and macrolide antibiotics predicted in 120 clinical E. coli isolates. Genes are sorted by mechanism into antibiotic efflux (purple), inactivation (orange), and target modification (green). Pie charts represent the total number of unique resistance genes predicted in the strains separated by their corresponding resistance mechanisms. (b) Histograms showing the distribution of rifampicin, novobiocin, and clarithromycin MICs in the presence and absence of SPR741. A dashed line marks the potentiation breakpoint concentration for each antibiotic.

OM perturbation reduces the MIC of potentiated antibiotics in a lab strain of E. coli harboring various antibiotic-inactivating resistance elements. We looked to investigate this phenotype using a collection of 120 E. coli isolates from a diverse range of tissues (blood, urine, rectal, and sputum) collected from patients in Ontario, Canada. We examined the impact of OM perturbation by SPR741 on the MICs of rifampicin, clarithromycin, and novobiocin. Each isolate was sequenced and analyzed for genes conferring resistance to rifamycin, aminocoumarin, and macrolide antibiotics (Fig. 3a and Table S2) using the Resistance Gene Identifier (RGI) software of the Comprehensive Antibiotic Resistance Database (CARD), which predicts the presence of resistance genes based on homology and single nucleotide polymorphism (SNP) models (28). This analysis indicated three mechanistic subtypes of resistance elements across isolates: efflux, antibiotic inactivation, and target modification. When classified with respect to antibiotic class, we found that the RGI predicted solely broad-spectrum efflux pumps to be putatively linked to rifamycin and aminocoumarin resistance. In contrast, inactivation and target modification resistance elements appeared to be macrolide specific (Fig. 3a and Table S2).

10.1128/mBio.01615-20.7 TABLE S2 Resistance genes predicted within 120 E. coli isolates using the Resistance Gene Identified (RGI) software of the Comprehensive Antibiotic Resistance Database (CARD). The presence of a gene is represented by 1 and the absence by 0. Download Table S2, XLSX file, 0.02 MB. Copyright © 2020 MacNair and Brown. 2020 MacNair and Brown This content is distributed under the terms of the Creative Commons Attribution 4.0 International license.

To determine whether OM perturbation could sensitize these strains to concentrations of the partner antibiotic theoretically obtainable during standard antibiotic treatment, we looked to assign a cutoff value similar to a traditional clinical breakpoint. Clinical breakpoint is conventionally defined as the concentration of antibiotic that defines a species of bacteria as susceptible or resistant. Breakpoint values for Gram-negative pathogens are not available for the traditionally Gram-positive active antibiotics used alongside OM perturbants. Therefore, we assigned a value deemed “potentiation breakpoint” to our antibiotic partners using the CLSI breakpoint value for the treatment of all *Staphylococcus* species. The selected potentiation breakpoints for rifampicin and clarithromycin are 1 μg/ml and 2 μg/ml, respectively. With the removal of novobiocin from the market in 2011, there is no currently listed clinical breakpoint. However, we considered a concentration of novobiocin as below the potentiation breakpoint when the MIC is less than steady-state serum levels (5 μg/ml) (34).

We first determined the MIC_50_ (MIC at which 50% of the isolates tested are inhibited) and MIC_90_ (MIC at which 90% of the isolates tested are inhibited) values for rifampicin, clarithromycin, and novobiocin in all 120 E. coli clinical isolates (Table 1). Without OM perturbation, MICs are above the potentiation breakpoint in all strains, while the addition of SPR741 reduced MIC_90_ values to below our potentiation breakpoint for rifampicin, clarithromycin, and novobiocin. The average reductions in MIC by the addition of SPR741 for rifampicin, novobiocin, and clarithromycin were 561, 162, and 551, respectively (Fig. 3b and Table S3). Potentiation below our selected breakpoint was observed for 118 of 120 strains in both rifampicin and novobiocin. Notably, the two resistant strains, C0004 and C0244, resisted potentiation by SPR741 for both novobiocin and rifampicin. Upon further investigation, these two strains were found to be resistant to OM perturbation by SPR741. The C0244 strain was highly resistant to polymyxin B, which is known to confer cross-resistance to the OM disruption by polymyxin derivatives similar to SPR741 (10). However, C0004 was sensitive to polymyxin B, and the mechanism behind the observed resistance to potentiation by SPR741 is currently unknown. Outside of strains resistant to OM perturbation, the MICs of novobiocin and rifampicin were reduced to clinically obtainable levels in all remaining isolates. Altogether, we would predict 118 of 120 strains to be susceptible to treatment by an OM perturbant combined with rifampicin or novobiocin, making these antibiotics highly attractive partners.

**TABLE 1:** MIC_50_ and MIC_90_ values for rifampicin, clarithromycin, and novobiocin in the presence and absence of SPR741 against E. coli (*n* = 120)

Antibiotic	E. coli		E. coli *+* SPR741	
	MIC_50_ (μg/ml)	MIC_90_ (μg/ml)	MIC_50_ (μg/ml)	MIC_90_ (μg/ml)
Rifampicin	3.125	6.25	0.01	0.04
Clarithromycin	50	400	0.1	1.56
Novobiocin	37.5	100	0.39	1.56

10.1128/mBio.01615-20.8 TABLE S3 Potency analysis of novobiocin, rifampicin, and clarithromycin in the presence and absence of SPR741 at 1/4 MIC. Fold reduction of MIC was determined by dividing the MIC of the antibiotic alone by its MIC with SPR741. Download Table S3, XLSX file, 0.02 MB. Copyright © 2020 MacNair and Brown. 2020 MacNair and Brown This content is distributed under the terms of the Creative Commons Attribution 4.0 International license.

**FIG 4:**
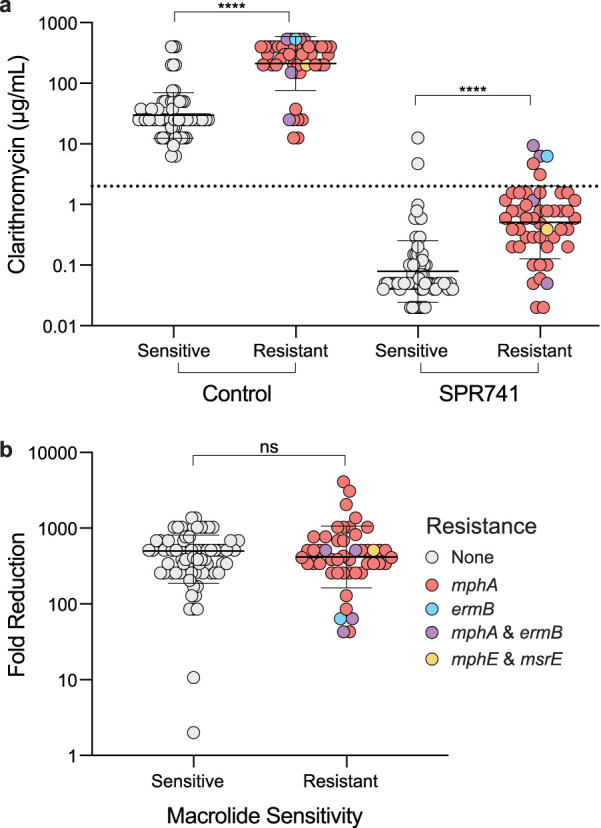
OM perturbation overcomes horizontally acquired macrolide resistance. (a and b) E. coli strains were divided into two groups. Strains predicted to contain macrolide-specific resistance elements (resistant [red, blue, purple, orange]) and no macrolide-specific resistance (sensitive [gray]). Resistant strains are subdivided by color into their predicted resistance genes. (a) MIC values of clarithromycin were significantly increased (****, *P* < 0.0001 by Mann-Whitney U test) in strains predicted to harbor macrolide resistance (Resistant) in the presence and absence of SPR741. The dotted line marks the potentiation breakpoint value of 2 μg/ml. (b) Fold reduction of clarithromycin MIC was not significantly different (ns) between the sensitive and resistant strains (*P* > 0.05, Mann-Whitney U test).

Given the large quantity of macrolide-specific resistant elements within our E. coli isolates, we aimed to examine their impact on the potentiation of clarithromycin in depth. Forty-eight strains were predicted to harbor at least one of the following macrolide resistance genes: *mphA*, *mphE*, *msrE*, or *ermB* (Table S2). We divided strains into two categories based on the presence or absence of macrolide-specific resistance elements. Strains harboring macrolide resistance (*mphA*, *mphE*, *msrE*, or *ermB*) were deemed “resistant,” and all other strains were deemed “sensitive” (Fig. 4a and b). We then monitored growth in the presence of clarithromycin with and without SPR741, finding a significant difference in MIC of “resistant” compared to “sensitive” isolates in both conditions (Fig. 4a). However, we observed no significant change in the range of fold reduction in MIC when comparing “resistant” and “sensitive” isolates (Fig. 4b). Importantly, SPR741 reduced the clarithromycin MIC to below the potentiation breakpoint in 113 of 120 clinical isolates (Fig. 3b, Fig. 4a, and Table S3).

We took particular interest in the seven strains where we were unable to reach the potentiation breakpoint of clarithromycin. Two strains in this group, C0004 and C0244, were not predicted to be macrolide-resistant. However, we previously identified these strains as having reduced susceptibility to OM disruption by SPR741. Three of the five remaining strains were predicted to contain *ermB*, a 23S rRNA methyltransferase similar to *ermC*, and were not potentiated below the breakpoint (Fig. 4b, Table S2): C0012, C0013, and C0452. Strains C0012 and C0452 contained both *mphA* and *ermB*, which may also contribute to the observed high level of resistance. Two strains, C0240 and C0008, were predicted to harbor *mphA* but no other macrolide-specific resistance elements. Despite the high frequency of predicted broad-spectrum and macrolide-specific resistance present in Gram-negative pathogens, the degree of potentiation is mostly unaffected (Fig. 4b), and the majority of MICs are reduced below the potentiation breakpoint (Table 1 and Fig. 3b).

The three mechanistic subtypes of resistance proved to each uniquely influence potentiation by OM perturbation. Broad-spectrum efflux pumps did not provide a barrier to potentiation below the breakpoint for rifampicin, novobiocin, or clarithromycin. Macrolide inactivation by phosphatases was common within our isolates, predicted in 47 strains. Inactivation by *mphA* or *mphE* proved mostly surmountable by OM perturbation, with 87% of harboring strains potentiated to the potentiation breakpoint. Resistance by *ermB* proved challenging, with 60% of strains (3 of 5) remaining above the potentiation breakpoint. Notably, *msrE*, which protects the ribosome from inhibition by physically removing macrolides from their binding site (35, 36), was overcome in the one strain harboring this resistance (Fig. 4a). Overall, these results are in concordance with the constitutively expressed resistance elements in a wild-type strain of E. coli (Fig. 2d), where antibiotic inactivation proved largely surmountable to OM perturbation and target modification was difficult to overcome.

We note here that the clinical strains used in this study do not cover a diverse geographic range, and regional differences in resistance prevalence may be encountered. For a more global perspective, we looked at the occurrence of macrolide resistance genes *mphA* and *ermB* in E. coli using 15,757 whole-genome sequence assemblies available from NCBI using CARD RGI software (28). E. coli is predicted to harbor m*phA* and *ermB* in 12.95% and 1.59% of strains, respectively. The relatively low incidence of *ermB* is encouraging, and we anticipate the combination of an OM perturbant and clarithromycin to be highly efficacious irrespective of geographic location.

### OM perturbation reduces spontaneous resistance and biofilm formation.

**FIG 5:**
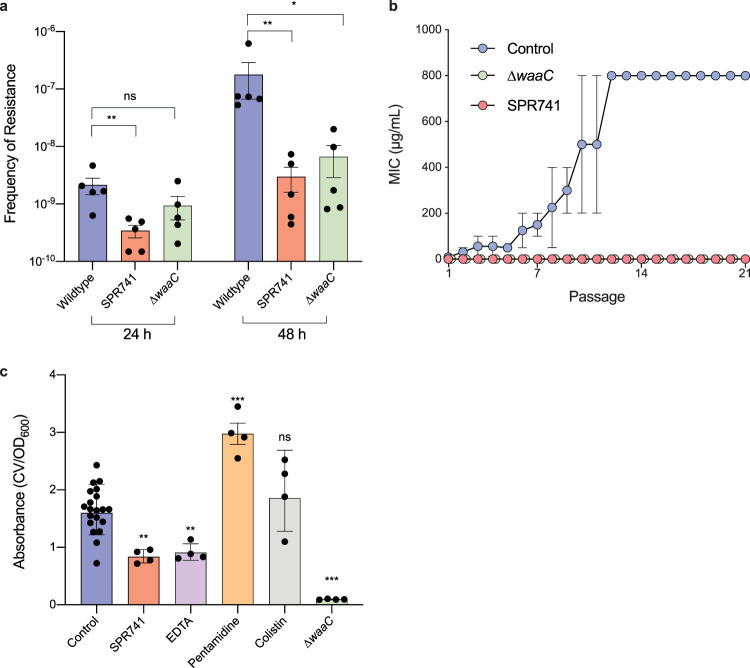
Disruption of the OM reduces spontaneous resistance to rifampicin and attenuates biofilm formation. (a) Frequency of resistance to rifampicin after 24 h and 48 h for E. coli, E. coli and SPR741, and E. coli Δ*waaC.* (b) Rifampicin MIC during serial passage of E. coli (blue), E. coli Δ*waaC* (green), and E. coli with SPR741 (red). (c) Crystal violet (CV) biofilm assay. Absorbance calculated by crystal violet (OD_570_)/growth (OD_600_). All data are shown with SEM and representative of at least two biological replicates. (c) Conditions are compared to the untreated control and significance calculated using the Mann-Whitney U test (ns, *P* ≥ 0.05; * *P* < 0.05; **, *P* < 0.01; ***, *P* < 0.001).

The combination of an OM perturbant and an otherwise inactive antibiotic partner requires the efficacy of both components to inhibit bacterial growth (22). As such, resistance may develop more rapidly than traditional monotherapy approaches (26, 27). Rifampicin was highly efficacious against our clinical E. coli strains (Table 1), providing significant therapeutic potential as a partner antibiotic. However, spontaneous resistance to rifampicin develops rapidly by mutations in *rpoB*, which reduce rifampicin binding to the ribosome (37). Indeed, as previously reported (22), this target modifying resistance was not overcome by OM perturbation (Fig. 2d). We determined the frequency of resistance (FOR) for rifampicin in the presence of OM perturbation by SPR741 and the deletion of *waaC.* After 24 h of incubation, E. coli displayed a resistance frequency of 2.13 × 10^−9^ (Fig. 5a). The addition of SPR741 significantly reduced the FOR to 3.42 × 10^−10^ compared to the E. coli control. Conversely, genetic perturbation (Δ*waaC*) did not significantly reduce the FOR with a mean FOR of 9.38 × 10^−10^. After 48 h of incubation, the FOR of E. coli increased 84-fold to 1.77 × 10^−7^. Comparatively, we observed only an ∼9-fold increase after 48 h with both SPR741 and Δ*waaC*, increasing FOR to 2.97.0 × 10^−9^ and 8.09 × 10^−9,^ respectively. We speculate that resistance at 24 h might represent the preexisting resistance in the population, while resistance after 48 h requires colonies to adapt to the antibiotic stress and develop resistance. Alternatively, this phenotype may simply be concentration-dependent, as we did not normalize rifampicin concentrations to the MIC of each condition.

We next examined the development of resistance by serial passaging, which controls for concentration dependence. Bacteria in three conditions, E. coli, E. coli with SPR741, and E. coli Δ*waaC*, were passaged for 21 days in rifampicin. Bacterial culture for each sequential passage was selected from the 1/4 MIC of the previous passage. In the control condition, E. coli rapidly gained resistance to rifampicin, with a 64-fold increase in MIC observed in just 12 passages to 800 μg/ml (Fig. 5b). However, passages in the presence of OM perturbation by SPR741 or Δ*waaC* showed only a fourfold or twofold increase in MIC, respectively, after 21 passages. The MIC of rifampicin remained below the potentiation breakpoint throughout the experiment. Overall, perturbation of the OM reduces spontaneous resistance development to rifampicin.

Biofilm formation poses a significant challenge in the treatment of bacterial infections. Gram-negative bacteria form biofilms composed predominantly of an extracellular polymeric substance (EPS) that contains anionic charge allowing association with divalent cations to provide stability (38). Noting the parallels between this interaction and the cation bridging of LPS, we speculated that OM disruption might impede biofilm formation. To address this, we assessed biofilm formation in E. coli alongside 1/4 MIC of the five OM perturbants EDTA, SPR741, colistin, pentamidine, and genetic deletion of *waaC.* The ablation of biofilm formation with EDTA (39, 40) and in E. coli Δ*waaC* (41) is consistent with previous reports. We did not identify a conserved impact on biofilm formation across OM perturbants; while both colistin and pentamidine trended toward an increase in biofilm formation, a significant reduction was observed for SPR741, EDTA, and Δ*waaC* (Fig. 5c). Despite the specificity of biofilm formation to individual perturbants, these results indicate that OM disruption by specific agents can reduce biofilm formation.

## DISCUSSION

Antibiotic development has failed to keep pace with the rapid dissemination of resistance. The impermeability of Gram-negative pathogens presents a unique challenge to discovery efforts. Disruption of the OM barrier through chemical or genetic perturbation can increase the susceptibility of Gram-negative bacteria to many traditionally Gram-positive active antibiotics. Several groups have published proof of principle studies for this approach using peptides (26), small molecules (10), chelators (19), and genetic perturbants (30). Despite a resurgence of effort in this area, previous work overwhelmingly focuses on the characterization of individual potentiator molecules, and the field lacks a thorough investigation of the strengths and limitations of this approach.

To better understand the changes in permeability conferred by OM perturbation, we screened a diverse library of bioactive molecules for potentiation with SPR741 and the deletion of *waaC*. In concordance with other reports (10, 15, 18), we found OM disruption to significantly increase E. coli susceptibility to hydrophobic compounds. In the presence of OM perturbation, hydrophobicity compatible with antimicrobial activity better correlated with MRSA than untreated E. coli. Expanding the range of physicochemical properties amenable to Gram-negative entry through OM perturbation has remarkable implications for antibiotic development. Indeed, accounts of biochemical screening and hit optimization efforts typical of modern target-based antibacterial drug discovery show that these efforts often produce potent inhibitors. However, the resulting compounds are invariably too hydrophobic and incompatible with Gram-negative entry (12). Our results suggest that a clinically viable OM disruptor would allow not only the immediate use of many Gram-positive active antibiotics but could also bring new life to previously abandoned drug leads.

The combination of an OM perturbant and Gram-positive active antibiotic requires the activity of both components to inhibit growth in Gram-negative bacteria. As such, resistance elements to antibiotics commonly partnered with OM perturbants have the potential to reduce combination efficacy. We discovered that the expression of antibiotic inactivation enzymes had minimal impact on the potentiation of erythromycin, clarithromycin, and rifampicin. However, resistance by target modification rendered potentiation by OM disruption largely ineffective. We speculate that inactivation enzymes may have a limited turnover rate that is being overwhelmed by increased antibiotic influx. However, target modifications, such as *rpoB*, can reduce the affinity of an antibiotic for its target by >1,000-fold (42), rendering increased influx ineffective at overcoming resistance.

Antibiotic inactivation and target modification resistance elements for Gram-positive active antibiotics are frequently harbored within Gram-negative pathogens. Looking to determine how this may impede the therapeutic potential of an OM perturbant, we investigated 120 clinical E. coli isolates for SPR741 potentiation of rifampicin, novobiocin, and clarithromycin. Genome analysis predicted nonspecific resistance by efflux machinery in all strains and no rifampicin- or novobiocin-specific resistance elements. MIC_90_ values for rifampicin and novobiocin were below the potentiation breakpoint in the presence of SPR741. Macrolide-specific resistance was predicted in 40% of the E. coli strains. Despite this, 113 of 120 strains were brought below the potentiation breakpoint, and typically, the presence of macrolide inactivation gene *mphA* or *mphB* did not render OM perturbation ineffective. Although target modification by *ermB* proved challenging to overcome, this resistance gene is predicted in less than 2% of global E. coli isolates, so we anticipate the use of clarithromycin alongside a potent OM perturbant to be efficacious against the overwhelming majority of E. coli strains.

Spontaneous resistance often reduces activity by modifying the antibiotic target, which as we have shown, can be difficult to overcome with OM perturbation. Additionally, bacteria have the opportunity to develop resistance to the OM-disrupting activity of the perturbant, potentially increasing the frequency of resistance. Therefore, we looked to determine whether OM perturbation alters the rate of spontaneous resistance. Rifampicin is particularly susceptible to spontaneous resistance, seemingly limiting its clinical potential for use alongside an OM perturbant. Direct plating of bacteria onto rifampicin with OM perturbation showed a reduced FOR that became more prominent over time. Serial passage experiments also showed a reduction in resistance development. The presence of SPR741 or the deletion of *waaC* was sufficient to maintain the MIC of rifampicin below the potentiation breakpoint for all 21 passages tested. We speculate that OM perturbation does not reduce preexisting resistance within a population but may impair the bacterium’s ability to adapt to antibiotic stress. Increased influx may reduce the time available to induce the SOS response, which is known to be essential for the development of rifampicin resistance (43).

Development of biofilms can severely impede antibiotic treatment for otherwise susceptible bacterial infections. Biofilms formed by Gram-negative pathogens are predominantly composed of EPS, which has many structural similarities to LPS. Therefore, we looked to determine whether chemical and genetic perturbations of the OM also impact biofilm formation. We observed variability in this phenotype across OM perturbants—EDTA, SPR741, and the genetic deletion of *waaC* reduced biofilm formation, while pentamidine and colistin increased biofilm formation. We reason that this increase may be attributed to the known ability for subinhibitory concentrations of antibiotics to stimulate biofilm formation (44, 45). That some OM perturbants impair biofilm formation is encouraging, particularly for guidelines that may be implemented to prioritize OM perturbants for further development.

In this work, we focused primarily on the impact of OM perturbation in E. coli. Previous efforts have indicated that the potentiation activity of OM perturbants is often conserved within *Enterobacteriaceae* species and A. baumannii (16). As such, we anticipate our results may be relevant to these pathogens also. Nevertheless, Pseudomonas aeruginosa is uniquely resistant to many OM perturbants, so we suggest further work may be in order to identify and investigate OM perturbants in this pathogen. We also note that our studies were conducted using high concentrations of each OM perturbant to ensure potent OM disruption. As it may be difficult to reliably reach comparable concentrations in patients using currently available OM perturbants, identifying more potent, nontoxic OM disruptors will surely be important to the therapeutic potential of this approach.

Targeting the OM is a unique and potentially revolutionary strategy for antibiotic discovery. OM perturbation sensitizes Gram-negative pathogens to a range of clinically approved Gram-positive active antibiotics and expands the chemical space compatible with novel antibiotic discovery efforts. However, many hurdles remain before this approach can be successfully implemented in the clinic. The selection or development of the correct OM perturbant and antibiotic partner combination will be instrumental in determining the success or failure of this approach. As with any combination therapy, optimization of dosing for sufficient overlap in bioavailability can prove difficult and requires more complex clinical trials than monotherapy approaches (46). Horizontally acquired resistance genes, spontaneous resistance development, and biofilms are all significant hurdles to successful antibiotic treatment. In this work, we uncover the capacity for OM disruption to overcome many of these challenges, uniquely positioning this approach among discovery efforts in the Gram-negative resistance crisis.

## MATERIALS AND METHODS

### Reagents.

SPR741 was provided by Spero Therapeutics. All other chemicals were purchased from Sigma-Aldrich. Compounds were routinely dissolved in dimethyl sulfoxide (DMSO) or H_2_O and stored at −20°C.

### Bacterial strains and culture conditions.

E. coli strain K-12 BW25113 or E. coli K-12 BW25113 (Δ*waaC*) was used for all experiments except those using the clinical isolate collection. The S. aureus USA300 JE2 strain was also used in compound screening. E. coli K-12 BW25113 was transformed with plasmids containing constitutively expressed resistance elements (47) (see Table S4 in the supplemental material). Clinical isolates of E. coli were collected from patients at Hamilton Health Sciences (HHS) hospital (Hamilton, Canada). Bacterial growth was in cation-adjusted Mueller-Hinton broth (MHB) at 37°C unless stated otherwise. Resistance gene prediction was conducted using CARD RGI software using paradigm “Strict” (28).

10.1128/mBio.01615-20.9 TABLE S4 Plasmids used to overexpress resistance elements in E. coli. Download Table S4, DOCX file, 0.01 MB. Copyright © 2020 MacNair and Brown. 2020 MacNair and Brown This content is distributed under the terms of the Creative Commons Attribution 4.0 International license.

### Potentiation assays.

All MICs were conducted in at least two biological replicates following the CLSI protocol (48). Fold reduction of MIC was determined by dividing the MIC of the antibiotic alone by its MIC in the treatment condition. OM probes in Fig. 1a were used at the following concentrations: EDTA (2 mM), colistin (0.05 μg/ml), pentamidine (75 μg/ml), and SPR741 (6.25 μg/ml). SPR741 was used at 6.25 μg/ml for all assays using laboratory E. coli. Potentiation assays for clinical E. coli isolates were conducted at 1/4 MIC SPR741.

### High-throughput compound screening.

All chemical screening was performed at the Centre for Microbial Chemical Biology (McMaster University). Overnight cultures of E. coli, E. coli Δ*waaC*, and S. aureus were brought to an optical density at 600 nm (OD_600_) of 0.1, diluted 1/200 into MHB for each condition tested, and dispensed into 384-well plates to a final volume of 30 μl per well. Sixty nl of each compound (5 mM stocks) was added for a final concentration of 10 μM per well. OD_600_ was read immediately after adding each compound and again after 18 to 20 h. Data were normalized by interquartile mean-based methods (49), and compounds reducing growth >50% were considered active compounds. Screening was performed in duplicate.

### Physicochemical property calculations.

Structure analysis was conducted using MarvinSuite 20.9.0, ChemAxon. Initial structure preparation was performed using the Standardizer tool to strip salts/solutes and verified with StructureChecker. Molecular weight and logD at pH 7.4 (cLogD) were then calculated using cxcalc.

### Biofilm formation assays.

Biofilm formation was determined in polystyrene 96-well plates as previously described (41) with minor changes. Briefly, bacteria were inoculated 1/500 from an overnight culture, and plates were prepared as in a standard MIC assay. After 48 h of incubation at 30°C, growth was measured by absorbance at OD_600_. Plates were then washed and dried at 37°C for 30 min, and crystal violet was added to the plates. After 30 min of incubation at room temperature, excess crystal violet was washed away, and the residual was solubilized with 30% acetic acid. Crystal violet was quantified by measuring OD_570_ and the relative amount of biofilm formed calculated by crystal violet (OD_570_)/growth (OD_600_). The concentrations of potentiators used are the same as in Fig. 1a.

### Resistant mutant development.

To conduct frequency of resistance (FOR) plating assays, an overnight culture of E. coli was diluted 1/500 into MHB and grown to mid-log phase (2 to 3 h), concentrated in phosphate-buffered saline (PBS), and 200 μl of cells was transferred onto solid MHB in 100-mm petri dishes supplemented with rifampicin (100 μg/ml) alone or rifampicin (100 μg/ml) and SPR741 (6.25 μg/ml). E. coli Δ*waaC* was plated only on rifampicin (100 μg/ml). Plating was also conducted on an SPR741 (6.25-μg/ml) control resulting in a lawn of bacteria after 24 h of incubation. Approximately 2 × 10^10^ CFU was deposited on each plate as determined by serial plating on nonselective MHB. Plates were incubated at 37°C, and resistant colonies were counted 24 h and 48 h postincubation. The frequency of resistance was calculated by dividing the number of resistant colonies by the total number of CFU plated. A subset of approximately 10 colonies per plate was selected and restreaked onto rifampicin or rifampicin with SPR741 to reconfirm resistance. All assays were conducted in biological duplicate, each composed of at least two technical replicates.

For passaging experiments, MICs of rifampicin were performed daily in three conditions, E. coli control, E. coli and SPR741 (6.25 μg/ml), or E. coli Δ*waaC*. MIC assays were performed as outlined above with the following modification: a 1/1,000 dilution of bacteria from the 1/4 MIC of the previous day’s passage was used to inoculate the subsequent passage. This process was continued for 21 passages in biological duplicate.
