# Supplementary figures and images for: Outer Membrane Disruption Overcomes Intrinsic, Acquired, and Spontaneous Antibiotic Resistance
Source: mBio. 2020 Sep 22;11(5):e01615-20. doi: 10.1128/mBio.01615-20 (PMC7512548; doi:10.1128/mBio.01615-20)

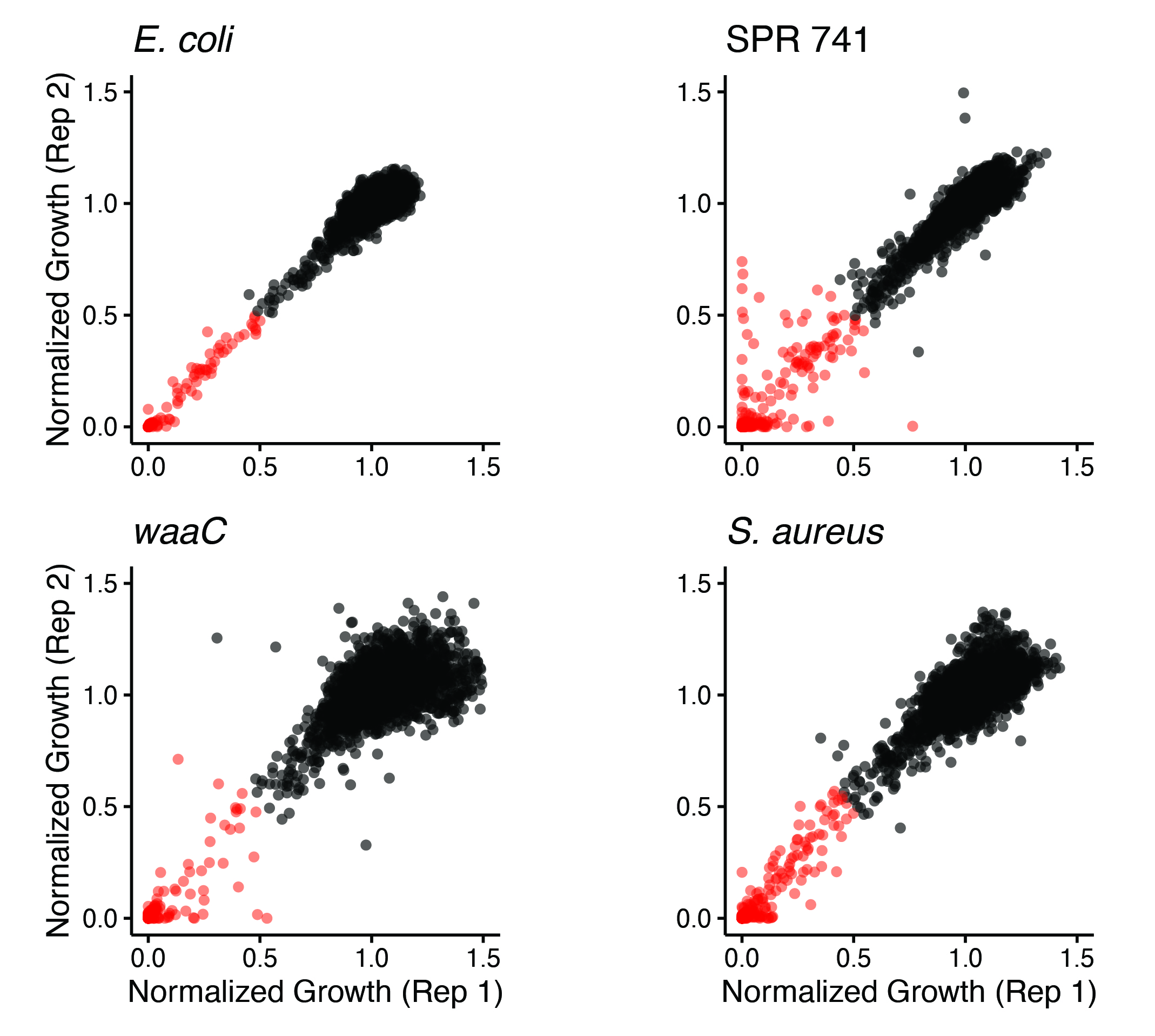

Supplement: FIG S1 [file mBio.01615-20-sf001.jpg]
