# Supplementary material for: Outer Membrane Disruption Overcomes Intrinsic, Acquired, and Spontaneous Antibiotic Resistance
Source: mBio. 2020 Sep 22;11(5):e01615-20. doi: 10.1128/mBio.01615-20 (PMC7512548; doi:10.1128/mBio.01615-20)
Supplement: TABLE S4 [file mBio.01615-20-st004.docx]

**Supplemental Table 4.** Plasmids used to overexpress resistance elements in *E. coli*.

| Antibiotic Class | Resistance Gene | Plasmid |
| --- | --- | --- |
| Macrolide | *mphA* | pGDP3 |
|  | *mphB* | pGDP3 |
|  | *ermC* | pGDP4 |
|  | *ereA* | pGDP2 |
| Rifamycin | *arr* | pGDP3 |
|  | *rph-Lm* | pGDP3 |
|  | *rpoB* | None |
| β-lactam | *bla_TEM-1_* | pGDP2 |
|  | *bla_VIM-2_* | pGDP1 |
|  | *bla_NDM-1_* | pGDP1 |
| Aminoglycoside | *aac(3)-Ia* | pGDP4 |
|  | *aph(4)-Ia* | pGDP3 |
| Tetracycline | *tetM* | pGDP2 |
|  | *tetR/tetA* | pGDP4 |
| Chloramphenicol | *CAT* | pGDP3 |
| Fosfomycin | *fosA* | pGDP1 |
| Fluroquinolone | *qnrA1* | pGDP2 |
